# A Two-Hybrid Assay to Study Protein Interactions within the Secretory Pathway

**DOI:** 10.1371/journal.pone.0015648

**Published:** 2010-12-28

**Authors:** Danielle H. Dube, Bin Li, Ethan J. Greenblatt, Sadeieh Nimer, Amanda K. Raymond, Jennifer J. Kohler

**Affiliations:** 1 Department of Chemistry and Biochemistry, Bowdoin College, Brunswick, Maine, United States of America; 2 Department of Chemistry, Stanford University, Stanford, California, United States of America; 3 Division of Translational Research, Department of Internal Medicine, University of Texas Southwestern Medical Center, Dallas, Texas, United States of America; 4 Biophysics Program, Stanford University, Stanford, California, United States of America; Johns Hopkins School of Medicine, United States of America

## Abstract

Interactions of transcriptional activators are difficult to study using transcription-based two-hybrid assays due to potent activation resulting in false positives. Here we report the development of the Golgi two-hybrid (G2H), a method that interrogates protein interactions within the Golgi, where transcriptional activators can be assayed with negligible background. The G2H relies on cell surface glycosylation to report extracellularly on protein-protein interactions occurring within the secretory pathway. In the G2H, protein pairs are fused to modular domains of the reporter glycosyltransferase, Och1p, and proper cell wall formation due to Och1p activity is observed only when a pair of proteins interacts. Cells containing interacting protein pairs are identified by selectable phenotypes associated with Och1p activity and proper cell wall formation: cells that have interacting proteins grow under selective conditions and display weak wheat germ agglutinin (WGA) binding by flow cytometry, whereas cells that lack interacting proteins display stunted growth and strong WGA binding. Using this assay, we detected the interaction between transcription factor MyoD and its binding partner Id2. Interfering mutations along the MyoD:Id2 interaction interface ablated signal in the G2H assay. Furthermore, we used the G2H to detect interactions of the activation domain of Gal4p with a variety of binding partners. Finally, selective conditions were used to enrich for cells encoding interacting partners. The G2H detects protein-protein interactions that cannot be identified via traditional two-hybrid methods and should be broadly useful for probing previously inaccessible subsets of the interactome, including transcriptional activators and proteins that traffic through the secretory pathway.

## Introduction

Identifying a protein's interaction partners is essential for deciphering protein function. The yeast two-hybrid (Y2H) system is a high-throughput genetic method that enables rapid genome-wide screening to discover a protein's interaction partners [1]. In its traditional and extensively-used form, the Y2H system relies on the ability of chimeric proteins to activate transcription of a reporter gene, an event that takes place in the nucleus. Although the Y2H is a powerful approach, limitations arise from the fact that protein-protein interactions are interrogated at a specific subcellular location, the nucleus. Transcriptional activators represent a class of proteins that cannot readily be studied using the traditional Y2H. These proteins can activate transcription on their own, thereby obscuring the transcriptional readout of the Y2H assay. For these reasons, an assay that examines protein-protein interactions at another subcellular location would provide a powerful complement to Y2H technology. Indeed, several groups have described two-hybrid or protein complementation assays (PCAs) that take place in the secretory pathway [2], [3], on the cell surface [4], or in the periplasmic space of bacteria [5], [6], but none of these methods has yet been widely adopted and each has its drawbacks. Those assays that rely solely on fluorescence-activated cell sorting [3], [4] necessitate access to special instrumentation. Furthermore, they are not as efficient at analyzing large libraries as assays that depend on survival or conditional growth. Assays that take place in bacteria [5], [6] may not be suitable for analyzing proteins that require specialized factors or post-translational modifications only present in mammalian cells.

To fill these gaps, we report the Golgi two-hybrid (G2H) system, a method for identifying protein-protein interactions in the secretory pathway of yeast. In the design of the G2H system, we took inspiration from key features of the traditional Y2H system. Specifically, we noted that the traditional Y2H system relies on the modularity of a transcription factor, which is separated into two domains. Protein-protein interactions bring together those two domains, reassembling the transcription factor and activating transcription of a reporter gene or genes. In an analogous fashion, our G2H method capitalizes on the modular nature of Golgi-resident glycosyltransferases, which consist of localization (LOC) and catalytic (CAT) domains. Golgi-resident glycosyltransferases add monosaccharides to protein and lipid substrates, a large fraction of which are subsequently trafficked to the cell surface or the extracellular space. In the G2H system, the interaction between two proteins reconstitutes the glycosyltransferase in a null background, thereby restoring wild-type cell surface glycosylation.

Selection and screening strategies are critical features of any approach to protein-protein interaction discovery. In the traditional Y2H system, a protein-protein interaction results in activation of a reporter gene that, in turn, confers a survival advantage or enables visual identification. Survival-based reporters, such as HIS3, provide selective pressure, thereby enabling rapid analysis of large libraries of cells. Survival-based selection can be combined with screening using a second reporter, such as LacZ, that induces a color or fluorescence change. By using two reporters, large cDNA libraries can be rapidly screened while eliminating many false positives. In contrast, most non-nuclear two-hybrid assays and PCAs rely on a single readout, thereby limiting throughput and increasing the incidence of false positives. Guided by the Y2H, we chose a Golgi-resident glycosyltransferase whose activity can be detected in multiple ways. In the G2H system, changes in cell surface glycosylation form the basis of both conditional growth and flow cytometry-based assays. Each of these assays could, in principle, be used both to screen individual strains and to select cells containing interacting bait-prey pairs from a mixed population.

We describe the fundamental features of the G2H method, a two-hybrid assay that takes place in the secretory pathway of eukaryotic cells. We demonstrate the utility of the G2H system by using it to observe protein-protein interactions that cannot be detected through traditional Y2H methods. We show that the selection strategies incorporated in the G2H method can be used to identify and enrich for cells containing interacting bait-prey pairs.

## Results

### The G2H reporter is the yeast glycosyltransferase Och1p

**Figure 1:**
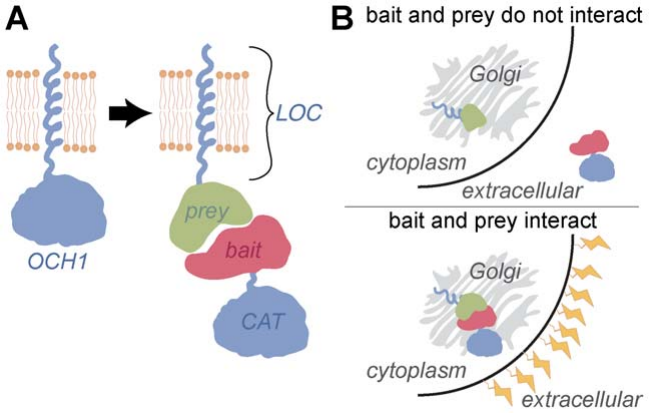
The Golgi two-hybrid assay is based on the modularity of the mannosyltransferase Och1p. (A) Och1p is predicted to be a type II transmembrane protein, with a single-pass transmembrane domain and a large, lumenal catalytic domain. Like other Golgi-resident glycosyltransferases, Och1p can be subdivided into localization (LOC) and catalytic (CAT) domains that function independently. When LOC and CAT are fused to interacting bait and prey, active Och1p is reassembled from two separate polypeptides. (B) When the bait and prey proteins do not interact, the bait-CAT is secreted to the outside of the cell and little Och1 activity is observed. When bait and prey do interact, bait-CAT is retained in the *cis* Golgi and the resulting Och1 activity causes a change in cell surface glycosylation.

The G2H assay is based on the reassembly of a Golgi-resident glycosyltransferase. Most Golgi-resident enzymes are composed of modular LOC and CAT domains [7], [8]. The N-terminal LOC domain dictates localization and is anchored in the membrane, while the C-terminal CAT domain resides in the lumen of Golgi and performs the sugar transfer reaction (Figure 1A) [9]. When these domains are expressed separately no catalytic activity is observed: the LOC domain is properly localized in the Golgi but lacks catalytic activity, and the CAT domain does not encounter its substrates because it is secreted by default [8]. Proteins to be interrogated, a bait and a prey, are then fused to the LOC and CAT fragments of a reporter glycosyltransferase (Figure 1). Interaction between bait and prey reconstitutes the glycosyltransferase and restores its activity, resulting in a concomitant change in cell surface glycosylation (Figure 1B).

**Figure 2:**
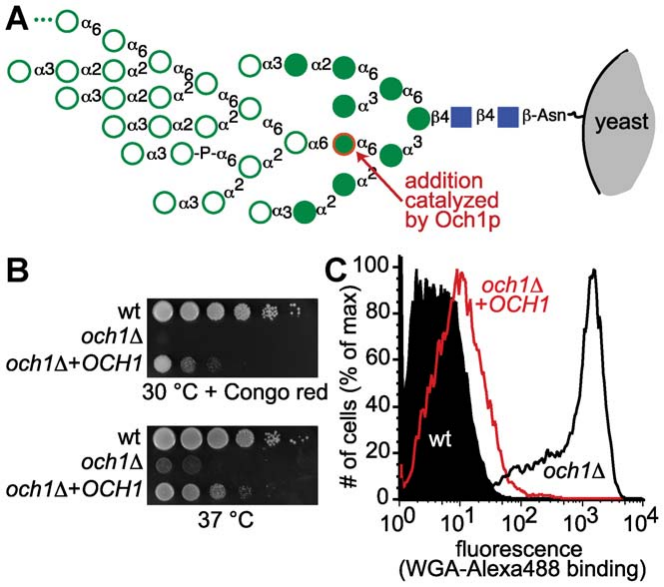
*och1*Δ yeast exhibit phenotypes that are reversed by *OCH1* expression. (A) Och1p activity is necessary for production of high-mannose glycans. Och1p catalyzes the addition of the α1-6-linked mannose residue highlighted in red. Once this sugar is added, other mannose monomers (shown by open circles) can be added. Further elaboration results in high-mannose glycans containing 50–200 monomer units. High-mannose glycans interfere with the ability of the WGA lectin to bind cell wall chitin. (B) *och1*Δ yeast grow slowly on agar plates when Congo red is present or when the temperature is elevated to 37°C. Introduction of a plasmid copy of *OCH1* partially restores growth. Each row shows ten-fold serial dilutions of the indicated strain. (C) Och1p activity can be inferred by measuring WGA binding by flow cytometry. Wild-type yeast bind WGA poorly, while *och1*Δ yeast bind WGA well. Expression of full-length *OCH1* in *och1*Δ complements the WGA binding phenotype.

Robust selection strategies are paramount to the utility of the G2H assay. Therefore, we sought to identify a glycosyltransferase whose activity could form the basis of a conditional growth assay as well as at least one complementary screening method. We examined phenotypic changes caused by the activity of Golgi-resident *S. cerevisiae* glycosyltransferases whose activities are critical to cell wall integrity [10], [11], [12], [13], [14] by assessing the growth of deletion strains at elevated temperature or in the presence of small molecule stressors. We focused attention on *och1*Δ because it grows only slightly slower than the parental strain under permissive conditions (30°C), but exhibits strong sensitivity to temperature and to Congo red, caffeine, and hygromycin (Figure S1). Och1p is an α1-6-mannosyltransferase that adds mannose to Man_8_GlcNAc_2_ in *N*-linked glycans to yield Man_9_GlcNAc_2_ [15]. This enzymatic reaction initiates the formation of the mannan outer chain. Once the product Man_9_GlcNAc_2_ is formed, a cascade of α1-6-mannosyltransferases acts to produce Man_50-100_GlcNAc_2_, a high mannose structure that covers the cell wall of wild-type yeast (Figure 2A). While *och1*Δ yeast exhibited dramatically slowed growth under non-permissive conditions (37°C or 30°C in the presence of Congo red), transformation with an *OCH1* plasmid rescued growth to close to wild-type levels (Figure 2B). Although *och1*Δ yeast exhibited reduced growth in both liquid culture (Figure S1) and on agar plates (Figure 2B), we conducted all subsequent growth assays on agar media because we were concerned that the flocculation tendency of *och1*Δ yeast [16] might interfere with our ability to accurately measure growth in liquid culture.

In addition to the conditional growth assays, we assessed the composition of *och1*Δ yeast cell walls by flow cytometry. Since Och1p plays a critical role in mannan biosynthesis, *och1*Δ yeast have dramatically different *N*-linked glycans from their wild-type counterparts. Chitin-binding reagents such as Alexa488-labeled lectin wheat germ agglutinin (WGA) bind strongly to *och1*Δ but weakly to the parental strain (Figure 2C). Furthermore, expression of full-length *OCH1* in *och1*Δ complements the WGA binding phenotype.

### The interaction between MyoD and Id2 restores Och1p activity

Our next step was to demonstrate that Och1p enzymatic activity could be reassembled from the component LOC and CAT domains. As in previous work [17], we used sequence alignment [18] of Och1p protein sequences from multiple species to identify the LOC and CAT domains. We designated *S. cerevisiae* Och1p amino acids 1-80 as LOC and Och1p amino acids 78-481 as CAT. DNA sequences encoding LOC and CAT domains were cloned into the yeast expression vectors p425-TEF and p426-TEF [19], respectively (Methods S1 and Tables S1 and S2). Since the catalytic domain contains no localization cue, we also included an N-terminal signal peptide to ensure its entry into the secretory pathway.

To evaluate the ability of Och1p activity to be reconstituted from its modular components, we examined a known protein-protein interaction, MyoD binding to Id2. While this interaction has been used as a positive control in Y2H assays, it presents a potential challenge for detection. In addition to forming a heterodimer with Id2, MyoD can also homodimerize through the same interface. We wondered if the G2H assay would be able to detect MyoD:Id2 complex formation in the face of competing MyoD homodimerization. Thus, we prepared plasmids encoding the fusion proteins LOC-MyoD and Id2-CAT. To ensure that any interaction we observed was specific, we also prepared a LOC domain fused to SV40 large T antigen (LOC-SV40TAg) and a CAT domain fused to p53 (p53-CAT).

**Figure 3:**
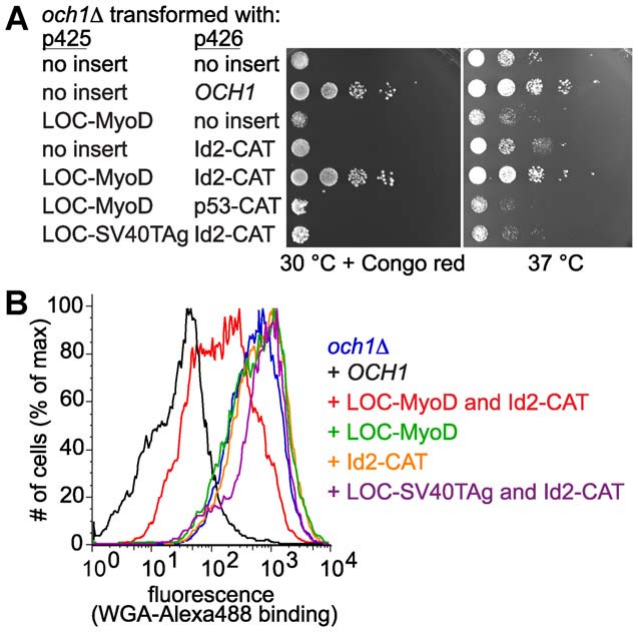
Interaction between MyoD and Id2 reassembles Och1p and reverses *och1*Δ phenotypes. (A) The temperature and Congo red sensitivity of *och1*Δ yeast can be rescued by co-expression of the interacting LOC-MyoD and Id2-CAT pair. *och1*Δ yeast were transformed with the indicated plasmids and grown on CSM-Leu-Ura agar plates at 30° in the presence of Congo red or at 37°C in the absence of Congo red. Each row shows ten-fold serial dilutions of the indicated strain. (B) Yeast strains were incubated with Alexa488-WGA and fluorescence was measured by flow cytometry. Decreased WGA binding is caused by co-expressing interacting LOC and CAT fusions (LOC-MyoD and Id2-CAT) in the *och1*Δ strain.

*och1*Δ yeast were transformed with pairs of plasmids. All strains grew at 30°C and only slight growth differences were apparent under these permissive growth conditions (Figure S2). Under non-permissive growth conditions (at 37°C or at 30°C in the presence of Congo red), dramatic differences in strain growth were observed (Figure 3A). *och1*Δ yeast transformed with either LOC-MyoD or Id2-CAT displayed *och1*Δ-like growth both at 37°C and at 30°C in the presence of Congo red (Figure 3A). Conversely, *och1*Δ co-transformed with both LOC-MyoD and Id2-CAT plasmids grew robustly at elevated temperature and on Congo red plates (Figure 3A). The observed growth differences are unlikely to be due to toxicity of the expressed proteins, because none of the plasmids affected growth of the parental yeast strain (Figure S3). We interpret the rescued growth observed in *och1*Δ[LOC-MyoD][Id2-CAT] yeast to mean that Och1p activity is reconstituted in these cells. The distinction between *och1*Δ and *och1*Δ[LOC-MyoD][Id2-CAT] was most pronounced in the presence of Congo red, suggesting that Congo red provides a stronger selective pressure than elevated temperature. Taken together, these data indicate that Och1p, like mammalian glycosyltransferases, is modular and can be reassembled into an active form via the interaction between MyoD and Id2.

To confirm that the growth phenotype observed in LOC-MyoD/Id2-CAT-transformed cells is due to Och1p activity, Alexa488-modified WGA lectin was employed to fluorescently label yeast based on cell wall composition. Flow cytometry analysis of yeast incubated with Alexa488-WGA revealed that the cell wall of *och1*Δ[LOC-MyoD][Id2-CAT] is shifted to resemble that of complemented *och1*Δ[OCH1], whereas the cell wall of *och1*Δ transformed with only Id2-CAT is indistinguishable from that of *och1*Δ cells (Figure 3B). These data provide further evidence that Och1p activity is present in cells containing both LOC-MyoD and Id2-CAT, yet is absent from cells containing only the Id2-CAT fusion. Thus, Och1p is modular, and its inactive modules can be reassembled via the MyoD-Id2 interaction to form a functional enzyme.

### Mutations to the MyoD-Id2 interaction interface increase Congo red sensitivity and WGA binding

To confirm that the rescued growth and WGA binding phenotypes observed in *och1*Δ[LOC-MyoD][Id2-CAT] yeast are due to the interaction between MyoD and Id2, we designed mutations to disrupt this interaction. Because a crystal structure of the MyoD-Id2 complex is not available, we modeled the MyoD-Id2 interaction using the crystal structure of a dimeric form of MyoD [20]. We reasoned that mutating the amino acids that form the tightly packed helix-helix contacts would interfere with MyoD:Id2 complex formation, preventing reassembly of Och1p and leading to increased sensitivity to Congo red and increased WGA binding. To test this possibility, we constructed plasmids that harbor point mutations in the second helix of the HLH domain of MyoD (Figure S4 and Table S3). These mutations encode changes that replace hydrophobic residues with lysines. We also prepared plasmids in which one or more turns of the second helix of MyoD are deleted.

**Figure 4:**
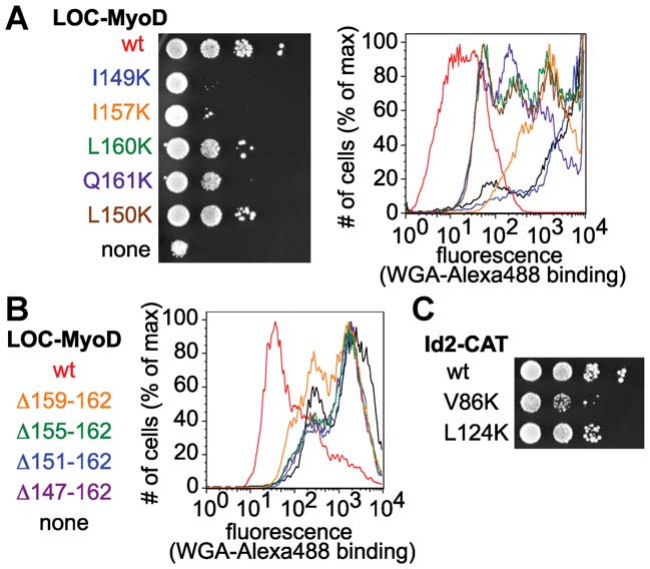
Mutations to MyoD and Id2 cause increases in Congo red sensitivity and WGA binding. (A) *och1*Δ yeast were transformed with wild-type Id2-CAT and LOC-MyoD plasmids containing point mutations to amino acids in the HLH domain of MyoD. Yeast were grown on CSM-Leu-Ura plates in the presence of Congo red. Each row shows ten-fold serial dilutions of the indicated strain. Yeast were also analyzed by flow cytometry using Alexa488-WGA. Strains expressing mutant LOC-MyoD plasmids exhibited slowed growth in the presence of Congo red and increased binding to WGA. (B) *och1*Δ yeast were transformed with wild-type Id2-CAT and with LOC-MyoD plasmids in which one or more turns of the interaction helix were deleted. Yeast were analyzed by flow cytometry using Alexa488-WGA. Strains expressing LOC-MyoD with deleted helices exhibited dramatically increased binding to WGA. (C) *och1*Δ yeast were transformed with LOC-MyoD and Id2-CAT plasmids containing point mutations to amino acids outside the HLH domain of Id2. Yeast were grown on CSM-Leu-Ura plates in the presence of Congo red. Each row shows ten-fold serial dilutions of the indicated strain. Strains expressing mutant Id2-CAT, instead of wild-type Id2-CAT, exhibited slightly slowed growth in the presence of Congo red.

As expected, *och1*Δ yeast expressing Id2-CAT and mutant forms of LOC-MyoD grew more slowly in the presence of Congo red than *och1*Δ yeast transformed with wild-type LOC-MyoD and Id2-CAT and demonstrated increased WGA binding (Figures 4A & 4B). Examination of the phenotypes observed for MyoD mutants suggests that the Congo red growth assay and the WGA binding assay may report on the relative strength of the bait-prey interaction. The I149K and I157K mutants produced severe growth defects and strong WGA binding. In fact, *och1*Δ yeast transformed with LOC-MyoD(I149K) and Id2-CAT were almost indistinguishable from *och1*Δ yeast transformed with Id2-CAT alone. This observation is expected given the very strong conservation of isoleucine at these positions throughout the bHLH family [21]. On the other hand, the L160K and Q161K mutations had a more modest effect, consistent with the fact that charged and polar amino acids are found at these positions in some bHLH family members [21]. In addition, L160 and Q161 are near the C-terminal end of the HLH motif and may not be as critical to packing as more centrally located residues. One surprise was the mild phenotypic changes observed for the L150K mutant, which appears to have only a small effect on the MyoD-Id2 interaction despite near conservation of leucine at this position throughout the bHLH family. As a control, we also made two point mutations to Id2, both of them outside of the canonical HLH motif: V86K is four amino acids C-terminal to the HLH motif, while L124K is 42 amino acids away. As expected, these mutations had milder effects than those made to the HLH region of MyoD and the severity of the phenotypes correlated with their proximity to the HLH motif (Figure 4C).

### The G2H detects interactions of Gal4p's activation domain (AD)

Next, we examined whether the G2H can detect interactions that cannot be studied using the traditional, transcription-based Y2H. The yeast transcription factor Gal4p contains a potent acidic AD that interferes with analysis by standard Y2H methods. *In vitro* affinity purification methods have been used to identify binding partners of Gal4p's AD, but the notoriously promiscuous binding properties of this domain have complicated analyses. As an alternative, Kodadek and colleagues used the Sos recruitment system [22] to demonstrate that the Gal4p AD interacts with Gal80p, Hap5p, and Rpt4p [23]. While successfully detecting some Gal4p AD binding partners, their analysis of a yeast cDNA library did not identify Gal11p, a known Gal4p AD binding partner. The absence of Gal11p was hypothesized to be due to the toxicity associated with overexpression of this transcriptional regulator, since Gal11 overexpression has been documented to cause a growth defect [24]. Taken together, the literature data suggested that detecting Gal4p's interactions would be a demanding test for our new assay.

**Figure 5:**
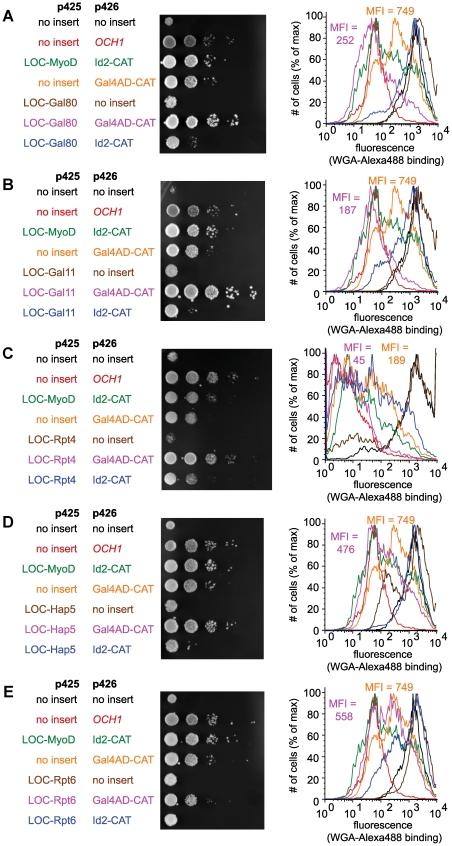
The Golgi two-hybrid assay detects interactions between a transcriptional activation domain and its binding partners. *och1*Δ yeast were transformed with Gal4AD-CAT and LOC-Gal80 (A), LOC-Gal11 (B), LOC-Rpt4 (C), LOC-Hap5 (D), or LOC-Rpt6 (E). Yeast were grown on CSM-Leu-Ura plates in the presence of Congo red. Each row shows ten-fold serial dilutions of the indicated strain. Yeast were also analyzed by flow cytometry using Alexa488-WGA. In each flow cytometry plot, the mean fluorescence intensity (MFI) is indicated for Gal4AD-CAT expressed alone and together with each LOC fusion. The observed increases in growth in the presence of Congo red and decreases in WGA binding provide strong evidence for Gal4AD interactions with Gal80p and Gal11p and somewhat weaker evidence for Gal4AD interactions with Rpt4p and Hap5p. The data do not support the Gal4AD-Rpt6p interaction.

To test the G2H's ability to detect Gal4p AD's interactions, *och1*Δ yeast were transformed with a plasmid encoding Gal4p AD fused to the *OCH1* CAT domain (Gal4AD-CAT) and with plasmids encoding the *OCH1* LOC domain fused to potential interaction partners. We observed that *och1*Δ yeast expressing Gal4AD-CAT alone exhibited increased growth in the presence of Congo red and decreased WGA binding, perhaps reflecting the notorious propensity of the acidic AD to interact nonspecifically with a variety of proteins [25]. Despite this increased background signal, we observed a significant growth enhancement and decrease in WGA binding when the yeast were co-transformed with Gal4AD-CAT and LOC-Gal80 (Figure 5A) or LOC-Gal11 (Figure 5B), and a more modest effect for yeast co-transformed with LOC-Rpt4 (Figure 5C) or LOC-Hap5 (Figure 5D). *och1*Δ yeast containing Gal4AD-CAT and LOC-Rpt6 demonstrated a slight decrease in WGA binding, but no significant change in growth phenotype relative to *och1*Δ yeast containing the Gal4AD-CAT construct alone (Figure 5E). These data suggest that the G2H can detect interactions of the Gal4p AD with Gal80p and Gal11p, and, to a lesser extent, Rpt4p and Hap5p. Our inability to detect an interaction between Gal4AD and Rpt6p is consistent with reported cross-linking data [26] and might indicate that these proteins do not interact directly. Using a non-transcriptional readout enabled us to examine the interactions of a transcriptional activator and moving interaction interrogation to the Golgi seems to have relieved the toxicity that is normally observed with Gal11p overexpression.

### Selective conditions enable enrichment of cells containing interacting bait-prey pairs

**Figure 6:**
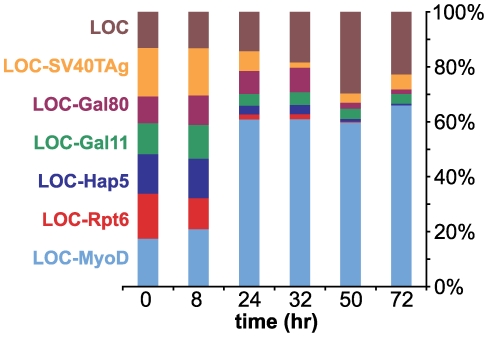
*och1*Δ yeast expressing LOC-MyoD and Id2-CAT outcompete strains expressing non-interacting LOC and CAT chimeras. Approximately equal quantities of strains expressing Id2-CAT and one of seven LOC plasmids were mixed together and cultured in media containing 10 mg/L Congo red. Quantitative PCR was used to measure the ratio of LOC plasmids present in the culture at different points in time. At 72 hr, the LOC-MyoD (66%) and LOC (23%) plasmids were dominant, while the LOC-Gal11 (4%), LOC-SV40TAg (5%), LOC-Hap5 (<1%), LOC-Gal80 (2%), and LOC-Rpt6 (<1%) plasmids were minor constituents of the mixture.

Having established that the G2H can be used to detect known protein-protein interactions, we wished to test whether the phenotypic changes that we observed could form the basis of a selection strategy. Approximately equal quantities of seven strains were mixed together and cultured under the selective pressure of Congo red. Each *och1*Δ strain was co-transformed with Id2-CAT and one of seven LOC plasmids: LOC (no fusion protein), LOC-SV40TAg, LOC-Gal80, LOC-Gal11, LOC-Hap5, LOC-Rpt6, or LOC-MyoD. The plasmid encoding LOC without a fusion protein was included to mimic typical plasmid library construction where some clones lack inserts. Strains were co-cultured for 72 hours and ratios of LOC plasmids in the culture were measured at various times using quantitative PCR. Within 24 hours, a significant enrichment of LOC-MyoD was observed (Figure 6). After 72 hours, the LOC-MyoD and LOC plasmids were dominant (66% and 23%, respectively) and all other LOC fusions were minor (<5%) constituents. These data indicate that, under selective conditions, yeast containing an interacting bait-prey pair rapidly outcompete those with non-interacting pairs. The representation of LOC alone increased slightly over time, while representation of all non-interacting LOC fusions decreased, suggesting that fusion of LOC to a non-interacting protein actually provides negative selective pressure. Based on these observations, we predict that the G2H method can be used to detect and enrich for yeast that harbor LOC and CAT constructs fused to novel interaction partners.

## Discussion

We describe the development of the G2H method and show that it can be used to detect interactions among proteins that cannot be studied using transcription-based two-hybrid systems, namely transcription factors. By virtue of its secretory pathway localization, the G2H also has the potential to be used to study the secretome, a class of proteins that remains poorly characterized [27]. Detecting protein interactions via the G2H method relies on robust phenotypic changes that will enable the G2H method to be used in both screening and selection experiments.

By interrogating protein-protein interactions in the Golgi, rather than the nucleus, the G2H provides a powerful complement to transcription-based approaches. We demonstrated two specific examples of the utility of Golgi localization. First, Gal4AD is a transcriptional activator and cannot easily be studied using assays that employ a transcriptional readout [23]. By testing Gal4p AD interactions in the Golgi, we avoided off-target transcriptional effects. Furthermore, we were to detect specific Gal4p interactions, even in the face of non-specific binding. Second, by targeting a toxic protein to the secretory pathway, we relieved its negative growth effects. Overexpression of Gal11p normally results in a dramatic decrease in growth [24], yet cells expressing the Golgi-localized LOC-Gal11 construct did not experience this toxicity. Indeed, the opposite was true: co-transformation with the LOC-Gal11 plasmid improved the growth of Gal4AD-CAT-expressing yeast.

In addition to detecting the interactions of Gal4AD with a number of binding partners, we were pleased to observe robust detection of the MyoD:Id2 interaction. Because the LOC-MyoD fusions are sequestered to the luminal face of the Golgi membrane, we were concerned that they might preferentially homodimerize with one another, rather than heterodimerizing with the soluble Id2-CAT fusion. Nonetheless, we are able to observe strong evidence of the LOC-MyoD:Id2-CAT heterodimeric interaction. Competition for binding will also be expected in cases where the prey is endogenously expressed within the Golgi and capable of competing with the LOC fusion protein for binding to the bait-CAT fusion protein. To investigate whether this situation will interfere with interaction detection by the G2H, we plan to conduct experiments to test the ability of the G2H to detect interactions that normally occur within the secretory pathway.

Like the traditional, transcriptional-based Y2H, the G2H is likely to have limitations. Proteins not normally localized to the secretory pathway could misfold in the G2H due to glycosylation of cryptic acceptor sequences or abnormal disulfide bond formation, thereby rendering them unable to engage in their normal protein-protein interactions. Misfolded proteins could also be retained in the ER, potentially leading to false positive signals. We observed reconstitution of Och1 in the Golgi through the MyoD:Id2 interaction and through the interactions of Gal4AD with a number of binding partners; therefore, at least some nuclear protein-protein interactions can assemble the secretory pathway environment, but others may be unable to do so due to differences in pH, ion concentration (Ca^2+^ in particular), oxidation state, or protein composition. Because the secretory environment is distinctly different from the nucleus, we predict that the G2H will be able to detect many protein-protein interactions that the classical, transcription-based Y2H cannot. Conversely, we expect that many interactions that are readily detected by the classical Y2H will be inaccessible to the G2H.

So far, the only case where the G2H assay failed to detect a well-characterized interaction was the p53:SV40TAg complex (data not shown). We speculate that the dodecameric structure of the SV40TAg:p53 complex [28] may be incompatible with the topology of the LOC and CAT fusions or that the high molecular weight complex interferes with correct trafficking of the Och1p fusion proteins, a phenomenon that was observed when another large oligomeric complex was ectopically localized to the secretory pathway [29]. If one of these hypotheses is correct, modifications to the G2H may be necessary to adapt it to analyses of proteins that oligomerize into very high molecular weight complexes. For example, inserting longer linkers between LOC and CAT domains and the bait and prey proteins may accommodate complex interaction geometry. Alternatively, using an ER-resident glycosyltransferase, rather than a Golgi-resident one, may enable detection of complexes that cannot exit the ER.

The experiments presented here describe a qualitative relationship between protein-protein interaction affinity and the signal observed in the G2H assay: the interaction between MyoD and Id2 produces strong signals, while introduction of mutations designed to disrupt this interaction decreases the strength of the phenotypic readouts. More comprehensive analysis will be needed to determine whether the signals observed in the growth and WGA binding assays are directly correlated with interaction affinity. A systematic analysis of interactions with varying affinities will enable us to answer this question and to assess the full dynamic range of this new assay.

The use of a glycosyltransferase, rather than a transcription factor, as a reporter enables new screening and selection methods. The Och1p reporter system described here relies on phenotypic changes observed in *och1*Δ yeast. The growth assay is simple to implement and its sensitivity can be adjusted by altering the concentration of Congo red. The WGA binding assay sensitively detects different levels of Och1p activity and, in principle, could be incorporated into a fluorescence-activated cell sorting (FACS) experiment to separate yeast with an active Och1p from those in which the protein is not reassembled.

By relying on a glycosyltransferase reporter, we envision that the G2H could also be adapted for use in other eukaryotic cells; all that is required is a modular reporter glycosyltransferase that causes a measurable cell surface change. Large families of glycosyltransferases occur in all eukaryotes, with 171 of these enzymes identified in humans [30]. In addition the yeast *Pichia pastoris* has recently been engineered to have human-like glycosylation patterns [31] and may have a secretory pathway better suited to discovering novel mammalian secretome protein-protein interactions [32]. For example, one could imagine using a G2H assay that incorporates human-like glycosylation to discover protein ligands for orphan cell surface receptors. Indeed, interactions among extracellular and cell surface proteins are poorly represented in existing protein-protein interaction databases [33] and new methods are needed to enable their discovery.

More broadly, glycosyltransferase activity has the potential to be more widely exploited for screening and selection experiments. The utility of glycosyltransferases stems from two key features. First, they are modular enzymes that can be reassembled from their component parts. Second, they have the ability to provide an extracellular report of intracellular events: the activity of secretory pathway glycosyltransferases occurs within the cell, but results in dramatic changes on the cell surface. In the same way that the transcription-based Y2H assay has been adapted to new uses, such as the discovery of protease substrates [34] and of protein-protein interactions that depend on post-translational modifications such as acetylation and phosphorylation [35], we anticipate the G2H has the potential to be used to report on biological events beyond simple protein-protein recognition.

## Materials and Methods

### Strains, plasmids, and growth conditions

*Saccharomyces cerevisiae* strains (MATa, background BY4741) were purchased from Open Biosystems. *S. cerevisiae* strains were grown on yeast extract, peptone and dextrose (YEPD) or on synthetic dextrose medium lacking leucine and uracil (CSM-Leu-Ura; MP Biomedicals). Detailed methods for plasmid construction are described in Methods S1. Primers used are shown in Tables S1, S2, and S3.

### Yeast transformation

All plasmids used are listed in Table S4. Plasmids were transformed into *och1*Δ yeast (background strain BY4741, MATa, his3Δ1, leu2Δ0, met15Δ0, ura3Δ0) or wild-type yeast (BY4741 MATa his3Δ1 leu2Δ0 met15Δ0 ura3Δ0) [36] by the lithium acetate/SS carrier DNA/PEG method [37] and selected on CSM-Leu-Ura plates. We used yeast colony PCR protocol to verify transformation. A single colony was transferred to 2 µL of sterile water in a microcentrifuge tube for each PCR reaction. The tubes were microwaved for 30 seconds and the contents used as a template for PCR. PCR cycling conditions were step 1: 94°C for 5 min; step 2: 94°C for 45 s; step 3: 60°C for 1 min; step 4: 72°C for 2 min; step 5: repeat steps 2-4 35 times; step 6: 72°C for 10 min. The yeast strains used are listed in Table S5.

### Yeast growth assays on agar plates

Growth of yeast strains on agar-based growth medium was scored by dilution plating. Yeast strains were grown in liquid culture at 30°C for two days in YEPD or SD-Leu-Ura, then standardized to an optical density at 600 nm (OD_600_) of 1.5. Strains were serially diluted ten-fold in media in a 96-well plate and then transferred to agar plates supplemented with or without Congo red (100 mg/L for YEPD plates; between 2.5 and 6 mg/L for CSM-Leu-Ura plates) using an inoculating manifold. Plates were incubated at 30°C or 37°C for 2 or 3 days and then imaged using an Alpha Innotech FluorChem HD2 photodocumentation system.

### Yeast growth assays in liquid culture

The ability of yeast strains to grow at 30°C or 37°C in liquid YEPD was measured by optical density. Starter cultures of wt, *och1*Δ, and *och1*Δ + *OCH1* were grown at 30°C for two days in YEPD. Fresh YEPD cultures were then inoculated (OD_600_ = 0.01) and grown for 3 days at 30°C or 37°C. The OD_600_ of each culture was measured in triplicate once daily for three days. Data were plotted as the average OD_600_ of each culture, with error bars representing +/− one standard deviation.

### Flow cytometry assay

Yeast cell wall chitin was detected using the lectin wheat germ agglutinin (WGA) and analyzed by flow cytometry. Yeast strains were grown at 30°C for two days in CSM-Leu-Ura, diluted to adjust OD_600_ to 0.4, then aliquoted into a 96-well plate with conical bottom (3 wells per strain; 200 µL per well). The yeast were pelleted (1200 *g* for 3 min in a tabletop centrifuge) and washed twice with 100 µL of FACS buffer (0.9% NaCl solution). Yeast were then incubated in 100 µL FACS buffer containing 0.01 or 0.02 mg/mL WGA-Alexa 488 (Invitrogen) for one hour at room temperature in the dark. After the incubation, yeast were washed three times with 100 µL of FACS buffer, then resuspended in 200 µL of FACS buffer and placed on ice. Flow cytometry experiments were performed on a FACSCalibur (BD Biosciences) instrument gating on 10,000 live cells per replicate. Data were analyzed using FlowJo software (Tree Star, Inc.). Representative data from each experiment are shown.

### Enrichment experiment

A small library was prepared by mixing together approximately equal quantities of seven *och1*Δ strains that were co-transformed with p426-Id2-CAT and one of seven p425-LOC plasmids: p425-LOC-MyoD, p425-LOC-SV40TAg, p425-LOC-Gal80, p425-LOC-Gal11, p425-LOC-Hap5, p425-LOC-Rpt6, and p425-LOC. The mix, having a starting OD_600_ of 0.2, was cultured in 400 mL CSM-Leu-Ura containing 10 mg/L Congo red. Yeast were harvested at 0, 8, 24, 32, 50, and 72 hr. Plasmid DNA was isolated the Wizard Plus SV Minipreps DNA Purification System (Promega), with the following modification to the manufacturer's protocol: after resuspending the cell pellet in the cell resuspension solution, the same volume acid-washed glass beads (Sigma) were added and mixture was vortexed for 3 min. The concentrations of plasmid DNA isolated for each time point were measured by UV absorbance.

Each qPCR reaction contained 10 µL of 2X iQ SYBR green Supermix (Bio-Rad), 1 µL of a 10 µM forward/reverse primer mix, 11.5 ng plasmid DNA and DNase/RNase-free water for a final volume of 20 µL. Cycling conditions were as follows: 95°C for 3.5 min, then 40 repeats of the following steps: 95°C for 30 sec, 60°C for 45 sec and 72°C for 2 min. SYBR green fluorescence was detected with a BioRad MyiQ2 Two-Color Real-Time PCR Detection System. Melting curves were obtained from 55°C to 98°C, with fluorescence measurements taken at every 0.5°C increase in temperature. Copy number of each plasmid was calculated by the iQ5 optical system software. Standard curves for primers and plasmids were obtained by 10-fold dilution of plasmids starting from 3,000,000 copies down to 300 copies. All reactions were carried out in triplicate and a non-template control was performed in each analysis.

## Supporting Information

Figure S1 **Yeast lacking mannosyltransferasases exhibit increased sensitivity to small molecule stressors.** (A) In liquid culture, yeast that lack *OCH1* exhibit a growth delay at 30°C and fail to grow at 37°C. Introduction of a plasmid copy of *OCH1* partially restores growth. (B) Wild-type yeast (BY4741) or mutants strains lacking mannosyltransferases were grown on YEPD with or without various small molecule stressors. *och1Δ* yeast exhibited strong sensitivity to caffeine, hygromycin, and Congo red. Each row shows ten-fold serial dilutions of the indicated strain.
(TIF)

Figure S2 **Under permissive conditions,** ***och1Δ*** **yeast transformed with LOC and CAT plasmids show only small growth differences.** *och1Δ* yeast transformed with the indicated plasmids were grown on CSM-Leu-Ura agar plates at 30°C in the absence of Congo red. Each row shows ten-fold serial dilutions of the indicated transformant.
(TIF)

Figure S3 **LOC and CAT plasmids are not toxic to yeast.** Wild-type yeast (BY4741) were transformed with the indicated plasmids and grown on CSM-Leu-Ura agar plates (A) at 30°C in the presence of 5 mg/L of Congo red or (B) at 37°C in the absence of Congo red. Each row shows ten-fold serial dilutions of the indicated transformant. In the context of this *OCH1*-expressing strain, the LOC and CAT constructs do not affect growth.
(TIF)

Figure S4 **Design of MyoD and Id2 mutations.** (A) Clustal (http://www.ebi.ac.uk/Tools/clustalw2/index.html) alignment of mouse MyoD and human Id2. The region of MyoD visible in the MyoD crystal structure is shaded in lavender and the portion of Id2 present in Id2-CAT is shaded in green. Sites of point mutations to MyoD and Id2 are indicated by red stars. All point mutations changed the native amino acid to lysine. The amino acids shown in purple, blue, green and gold represent regions of MyoD that were deleted in the helix deletion mutants. (B) Model of the interaction between the HLH regions of MyoD and Id2 was created based on the crystal structure of the dimeric MyoD bHLH domain bound to DNA (pdb code: 1MDY). Using the clustal alignment, the amino acids of the HLH region of one MyoD monomer were mutated to the corresponding Id2 residues. The modeled structure was rendered in PyMol and the sites of mutagenesis are highlighted. The terminal residues are labeled to provide reference to the sequence alignment.
(TIF)

Table S1 **Primers used in the localization domain constructs.** Sequence and names of primers used to prepare localization domain constructs. Restriction sites are underlined.
(DOC)

Table S2 **Primers used in the catalytic domain constructs.** Sequence and names of primers used to prepare catalytic domain constructs. Restriction sites are underlined.
(DOC)

Table S3 **Primers used for MyoD and Id2 mutagenesis.** Primers used for site-directed mutagenesis of MyoD- and Id2-encoding DNA sequences. In the case of point mutations, the mutated codon is shown in bold-faced type.
(DOC)

Table S4 **Plasmids used.** Names and descriptions of all plasmids used in this work.
(DOC)

Table S5 **Yeast strains used.** Descriptions of all yeast strains used in this work and the figures in which they were used.
(DOC)

Methods S1 **Plasmid construction.** Provides detailed description of the methods used for plasmid construction.
(DOC)
