# Supplementary figures and images for: A Two-Hybrid Assay to Study Protein Interactions within the Secretory Pathway
Source: PLoS One. 2010 Dec 28;5(12):e15648. doi: 10.1371/journal.pone.0015648 (PMC3011011; doi:10.1371/journal.pone.0015648)

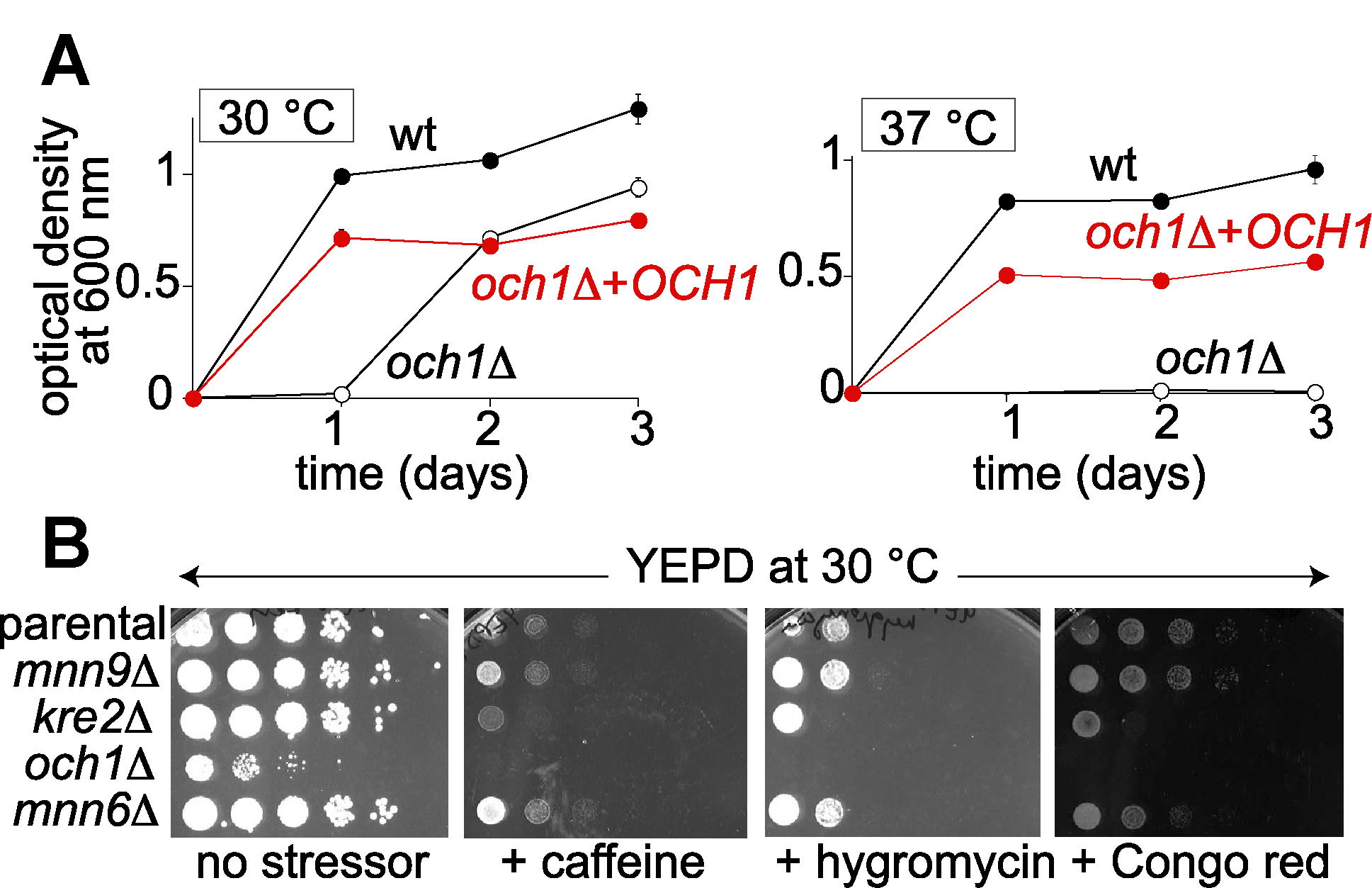

Supplement: Figure S1 — Yeast lacking mannosyltransferasases exhibit increased sensitivity to small molecule stressors. (A) In liquid culture, yeast that lack OCH1 exhibit a growth delay at 30°C and fail to grow at 37°C. Introduction of a plasmid copy of OCH1 partially restores growth. (B) Wild-type yeast (BY4741) or mutants strains lacking mannosyltransferases were grown on YEPD with or without various small molecule stressors. och1Δ yeast exhibited strong sensitivity to caffeine, hygromycin, and Congo red. Each row shows ten-fold serial dilutions of the indicated strain. (TIF) [file pone.0015648.s001.tif]

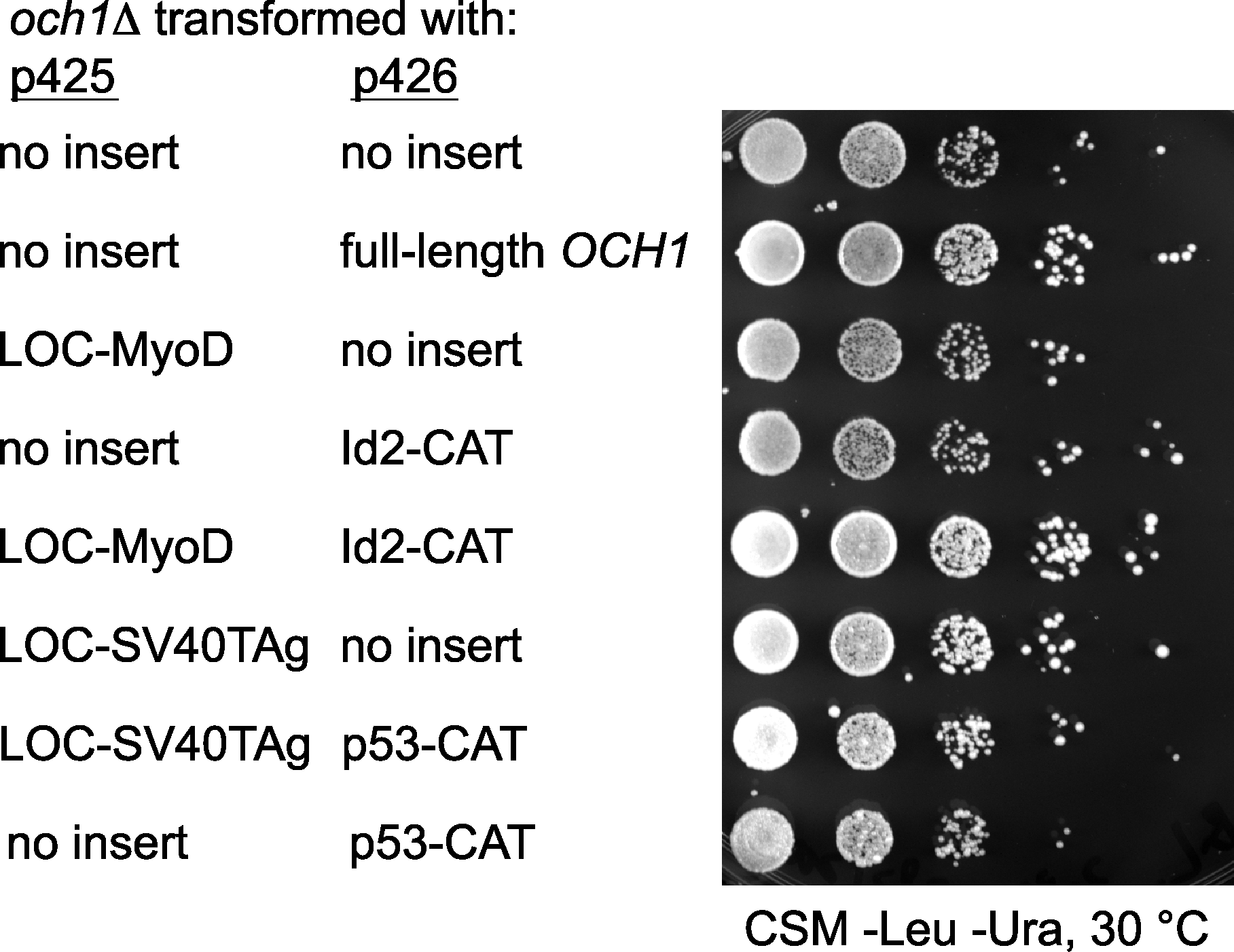

Supplement: Figure S2 — Under permissive conditions, och1Δ yeast transformed with LOC and CAT plasmids show only small growth differences. och1Δ yeast transformed with the indicated plasmids were grown on CSM-Leu-Ura agar plates at 30°C in the absence of Congo red. Each row shows ten-fold serial dilutions of the indicated transformant. (TIF) [file pone.0015648.s002.tif]

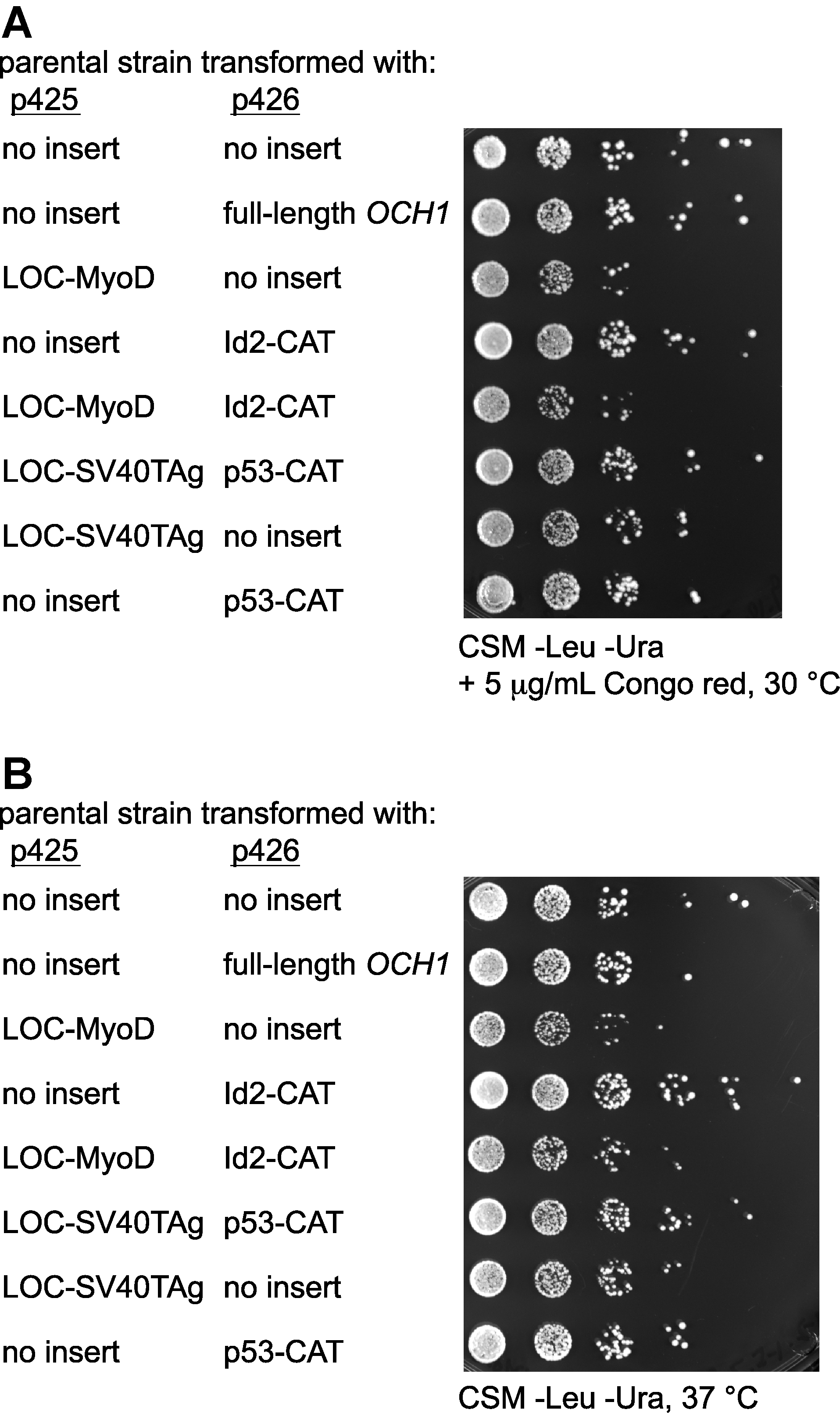

Supplement: Figure S3 — LOC and CAT plasmids are not toxic to yeast. Wild-type yeast (BY4741) were transformed with the indicated plasmids and grown on CSM-Leu-Ura agar plates (A) at 30°C in the presence of 5 mg/L of Congo red or (B) at 37°C in the absence of Congo red. Each row shows ten-fold serial dilutions of the indicated transformant. In the context of this OCH1-expressing strain, the LOC and CAT constructs do not affect growth. (TIF) [file pone.0015648.s003.tif]

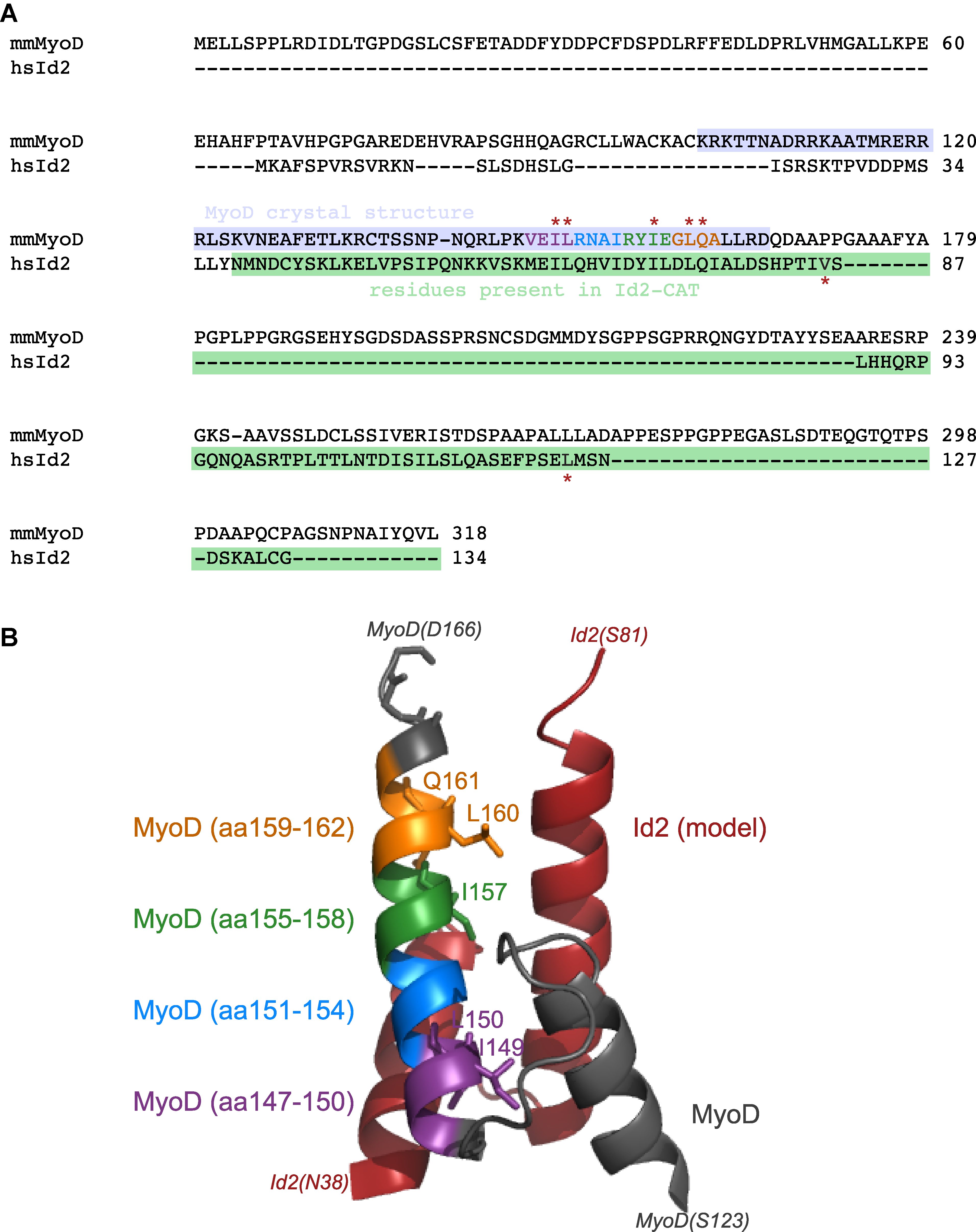

Supplement: Figure S4 — Design of MyoD and Id2 mutations. (A) Clustal (http://www.ebi.ac.uk/Tools/clustalw2/index.html) alignment of mouse MyoD and human Id2. The region of MyoD visible in the MyoD crystal structure is shaded in lavender and the portion of Id2 present in Id2-CAT is shaded in green. Sites of point mutations to MyoD and Id2 are indicated by red stars. All point mutations changed the native amino acid to lysine. The amino acids shown in purple, blue, green and gold represent regions of MyoD that were deleted in the helix deletion mutants. (B) Model of the interaction between the HLH regions of MyoD and Id2 was created based on the crystal structure of the dimeric MyoD bHLH domain bound to DNA (pdb code: 1MDY). Using the clustal alignment, the amino acids of the HLH region of one MyoD monomer were mutated to the corresponding Id2 residues. The modeled structure was rendered in PyMol and the sites of mutagenesis are highlighted. The terminal residues are labeled to provide reference to the sequence alignment. (TIF) [file pone.0015648.s004.tif]
