# Supplementary material for: A Two-Hybrid Assay to Study Protein Interactions within the Secretory Pathway
Source: PLoS One. 2010 Dec 28;5(12):e15648. doi: 10.1371/journal.pone.0015648 (PMC3011011; doi:10.1371/journal.pone.0015648)
Supplement: Table S1 — Primers used in the localization domain constructs. Sequence and names of primers used to prepare localization domain constructs. Restriction sites are underlined. (DOC) [file pone.0015648.s005.doc]

| **Supporting Table 1. Primers used in the localization domain constructs.** | | |
| --- | --- | --- |
| Primer Name | Sequence (5’ to 3’) | Restriction site |
| LOCF | GGACTAGTATGTCTAGGAAGTTGTCC | *SpeI* |
| LOCR | CGCGGATCCTAAATTATGCAATTGATTTTT | *BamHI* |
| LOCstopR | CGCGGATCC*TCA*TAAATTATGCAATTGATTTTT | *BamH*I |
| MyoDF | GGATCCGGACCTAGGGGACATATGATGGAGCTTCTATCGCCGCC |  |
| MyoDR | CTCGAGGCTAGCGGAGCATGCAAGCACCTGATAAATCGCATTGGG |  |
| SV40F | GGATCCGGACCTAGGGGACATATGACTGATGAATGGGAGCAGTGG |  |
| SV40R | CTCGAGGCTAGCGGAGCATGCTGTTTCAGGTTCAGGGGGAG |  |
| Gal80F | CGCGGATCCTGGACTACAACAAGAGATCTTC | *BamH*I |
| Gal80R | CCGCTCGAGTTATAAACTATAATGCGAGATATTGC | *Xho*I |
| Gal11F | CGCGGATCCATGTCTGCTGCTCCTGTCC | *BamH*I |
| Gal11R | CCGCTCGAGTCAAGCTTGGATTTTTCTCAGGGCCT | *Xho*I |
| Hap5F | CGCGGATCCATGACTGATAGGAATTTCTCACC | *BamH*I |
| Hap5R | CCGCTCGAGTCATTGTGGAAGAGGTCTTCTAG | *Xho*I |
| Rpt6F | CGCGGATCCATGACAGCTGCTGTAACATCCT | *BamH*I |
| Rpt6R | CCGCTCGAGTCACTTGAACAGCTTGGCGAC | *Xho*I |
| Rpt4F | CGCCCTAGGATGAGTGAAGAACAGGACC | *Avr*II |
| Rpt4R | CCGCTCGAGTCATAATTTTTGGTATTCTATAGTG | *Xho*I |
