# Supplementary material for: A Two-Hybrid Assay to Study Protein Interactions within the Secretory Pathway
Source: PLoS One. 2010 Dec 28;5(12):e15648. doi: 10.1371/journal.pone.0015648 (PMC3011011; doi:10.1371/journal.pone.0015648)
Supplement: Table S2 — Primers used in the catalytic domain constructs. Sequence and names of primers used to prepare catalytic domain constructs. Restriction sites are underlined. (DOC) [file pone.0015648.s006.doc]

| **Supporting Table 2. Primers used in the catalytic domain constructs.** | | |
| --- | --- | --- |
| Primer Name | Sequence (5’ to 3’) | Restriction site |
| CATF | CCGAAGCTTCATAATTTACGTGATCAATT | *Hind*III |
| CATR | CCGCTCGAGTTATTTATGACCTGCATTTTTAT | *Xho*I |
| ID2F | GGATCCGGACCTAGGAACATGAACGACTGCTACTC | *BamHI, Avr II* |
| ID2R | AAGCTTGCTAGCGGAGCATGCGCCACAGAGTACTTTGCTATCATTCGAC |  |
| P53F | GGATCCGGACCTAGGGGACATATGTGTCACCGAGACCCCTGG |  |
| P53R | AAGCTTGCTAGCGGAGCATGCGTCTGAGTCAGGCCCCAC |  |
| Gal4ADF | CGCGGATCCGACCAAACTGCGTATAACG | *BamH*I |
| Gal4ADR | CCCAAGCTTTGGGGTATCTTCATCATCG | *Hind*III |
