# Supplementary material for: A Two-Hybrid Assay to Study Protein Interactions within the Secretory Pathway
Source: PLoS One. 2010 Dec 28;5(12):e15648. doi: 10.1371/journal.pone.0015648 (PMC3011011; doi:10.1371/journal.pone.0015648)
Supplement: Table S3 — Primers used for MyoD and Id2 mutagenesis. Primers used for site-directed mutagenesis of MyoD- and Id2-encoding DNA sequences. In the case of point mutations, the mutated codon is shown in bold-faced type. (DOC) [file pone.0015648.s007.doc]

| **Supporting Table 3. Primers used for MyoD and Id2 mutagenesis.** | |
| --- | --- |
| Primer Name | Sequence (5’ to 3’) with codon to be mutated shown in **bold** |
| I149KF | GGCTACCCAAGGTGGAG**AAG**CTGCGCAACGCCATCCG |
| I149KR | CGGATGGCGTTGCGCAG**CTT**CTCCACCTTGGGTAGCC |
| L150KF | ACCCAAGGTGGAGATC**AAG**CGCAACGCCATCCGCT |
| L150KR | AGCGGATGGCGTTGCG**CTT**GATCTCCACCTTGGGT |
| I157KF | CAACGCCATCCGCTAC**AAG**GAAGGTCTGAAGGCTC |
| I157KR | GAGCCTTCAGACCTTC**CTT**GTAGCGGATGGCGTTG |
| L160KF | CCGCTACATCGAAGGT**AAG**CAGGCTCTGCTGCG |
| L160KR | CGCAGCAGAGCCTG**CTT**ACCTTCGATGTAGCGG |
| Q161KF | CATCGAAGGTCTG**AAG**GCTCTGCTGCGC |
| Q161KR | GCGCAGCAGAGC**CTT**CAGACCTTCGATG |
| ID2V85KF | GACTCGCATCCCACTATC**AAG**AGCCTGCATCACCAGAGAC |
| ID2V85KR | GTCTCTGGTGATGCAGGCT**CTT**GATAGTGGGATGCGAGTC |
| ID2L123KF | GCAGGCATCTGAATTCCCTTCTGAG**AAG**ATGTCGAATGATAGCAAAGTAC |
| ID2L123KR | GTACTTTGCTATCATTCGACAT**CTT**CTCAGAAGGGAATTCAGATGCCTGC |
| overlapD147F | CGAACCAGCGGCTACCCAAGCTGCTGCGCGACCAGGACG |
| overlapD147R | CGTCCTGGTCGCGCAGCAGCTTGGGTAGCCGCTGGTTCG |
| overlapD151F | GCTACCCAAGGTGGAGATCCTGCTGCTGCGCGACCAGGACG |
| overlapD151R | CGTCCTGGTCGCGCAGCAGCAGGATCTCCACCTTGGGTAGC |
| overlapD155F | AGATCCTGCGCAACGCCATCCTGCTGCGCGACCAGGACG |
| overlapD155R | CGTCCTGGTCGCGCAGCAGGATGGCGTTGCGCAGGATCT |
| overlapD159F | CAACGCCATCCGCTACATCGAACTGCTGCGCGACCAGGACG |
| overlapD159R | CGTCCTGGTCGCGCAGCAGTTCGATGTAGCGGATGGCGTTG |
