# Supplementary material for: A Two-Hybrid Assay to Study Protein Interactions within the Secretory Pathway
Source: PLoS One. 2010 Dec 28;5(12):e15648. doi: 10.1371/journal.pone.0015648 (PMC3011011; doi:10.1371/journal.pone.0015648)
Supplement: Table S4 — Plasmids used. Names and descriptions of all plasmids used in this work. (DOC) [file pone.0015648.s008.doc]

| **Supporting Table 4. Plasmids used** | |
| --- | --- |
| *Plasmid name* | *Source* |
| p425-Empty | p425-TEF (supporting ref. 1) |
| p426-Empty | p426-TEF (supporting ref. 1) |
| *Plasmid name* | *Gene encoded* |
| p425-Loc-MyoD | OCH1(1-80)-MyoD(1-318) |
| p425-Loc-Sv40 | OCH1(1-80)-SV40TAg(88-708) |
| p425-Loc-MyoD(I149K) | OCH1(1-80)-MyoD(1-318)(I149K) |
| p425-Loc-MyoD(I157K) | OCH1(1-80)-MyoD(1-318)(I157K) |
| p425-Loc-MyoD(L160K) | OCH1(1-80)-MyoD(1-318)(L160K) |
| p425-Loc-MyoD(Q161K) | OCH1(1-80)-MyoD(1-318)(Q161K) |
| p425-Loc-MyoD(L150K) | OCH1(1-80)-MyoD(1-318)(L150K) |
| p425-Loc-MyoD(Δ159-162) | OCH1(1-80)-MyoD(1-318) (Δ159-162) |
| p425-Loc-MyoD(Δ155-162) | OCH1(1-80)-MyoD(1-318) (Δ155-162) |
| p425-Loc-MyoD(Δ151-162) | OCH1(1-80)-MyoD(1-318) (Δ151-162) |
| p425-Loc-MyoD(Δ147-162) | OCH1(1-80)-MyoD(1-318) (Δ147-162) |
| p425-Loc-Gal80 | OCH1(1-80)-Gal80(1-435) |
| p425-Loc-Gal11 | OCH1(1-80)-Gal11(1-351) |
| p425-Loc-Rpt4 | OCH1(1-80)-Rpt4(1-438) |
| p425-Loc-Hap5 | OCH1(1-80)-Hap5(1-243) |
| p425-Loc-Rpt6 | OCH1(1-80)-Rpt6(1-406) |
| P425-Loc(stop codon) | OCH1(1-80)-Stop codon |
| p426-full length och1 | OCH1(1-481) |
| p426-Id2-Cat | Id2(38-137)-OCH1(78-481) |
| p426-p53-Cat | p53(125-390)-OCH1(78-481) |
| p426-Id2(V86K)-Cat | Id2(38-137)(V86K)-OCH1(78-481) |
| p426-Id2(L124K)-Cat | Id2(38-137)(L124K)-OCH1(78-481) |
| p426-Gal4AD-Cat | Gal4 (841-874)-OCH1(78-481) |
