# Supplementary material for: A Two-Hybrid Assay to Study Protein Interactions within the Secretory Pathway
Source: PLoS One. 2010 Dec 28;5(12):e15648. doi: 10.1371/journal.pone.0015648 (PMC3011011; doi:10.1371/journal.pone.0015648)
Supplement: Table S5 — Yeast strains used. Descriptions of all yeast strains used in this work and the figures in which they were used. (DOC) [file pone.0015648.s009.doc]

| **Supporting Table 5. Yeast strains used** | | |
| --- | --- | --- |
| *Yeast strains* | *Genotype* | |
| och1Δ | BY4741 MATa his3Δ1 leu2Δ0 met15Δ0 ura3Δ0 och1Δ (ref. 36) | |
| Wild type | BY4741 MATa his3Δ1 leu2Δ0 met15Δ0 ura3Δ0 (ref. 36) | |
|  | | |
| *Background strain* | *Plasmids transformed* | *Used in* |
| och1Δ | p425-Empty + p426-Empty | Fig3A, 5A, 5B, 5C, 5D, 5E, S2 |
| och1Δ | p425-Empty + p426-full length *OCH1* | Fig3A, 5A, 5B, 5C, 5D, 5E, S2 |
| och1Δ | p425-Empty + p426-Id2Cat | Fig3A, 4A, 4B, S2 |
| och1Δ | p425-Loc-MyoD + p426-Empty | Fig3A, S2 |
| och1Δ | p425-Loc-MyoD+ p426-Id2-Cat | Fig3A, 4A, 4B, 4C, 5A, 5B, 5C, 5D, 5E, 6, S2 |
| och1Δ | p425-Loc-MyoD+p426-p53-Cat | Fig3A |
| och1Δ | p425-Loc-SV40TAg+p426-p53-Cat | FigS2 |
| och1Δ | p425-Loc-SV40TAg+p426-Id2-Cat | Fig3A |
| och1Δ | p425-Loc-MyoD(I149K)+p426-Id2-Cat | Fig4A |
| och1Δ | p425-Loc-MyoD(I157K)+p426-Id2-Cat | Fig4A |
| och1Δ | p425-Loc-MyoD(I160K)+p426-Id2-Cat | Fig4A |
| och1Δ | p425-Loc-MyoD(I161K)+p426-Id2-Cat | Fig4A |
| och1Δ | p425-Loc-MyoD(I150K)+p426-Id2-Cat | Fig4A |
| och1Δ | p425-Loc-MyoD(Δ159-162)+p426-Id2-Cat | Fig4B |
| och1Δ | p425-Loc-MyoD(Δ155-162)+p426-Id2-Cat | Fig4B |
| och1Δ | p425-Loc-MyoD(Δ151-162)+p426-Id2-Cat | Fig4B |
| och1Δ | p425-Loc-MyoD(Δ147-162)+p426-Id2-Cat | Fig4B |
| och1Δ | p425-Loc-MyoD+ p426-Id2(V86K)-Cat | Fig4C |
| och1Δ | p425-Loc-MyoD+ p426-Id2(L124K)-Cat | Fig4C |
| och1Δ | p425-Empty+p426-Gal4AD-Cat | Fig5A, 5B, 5C, 5D, 5E |
| och1Δ | p425-Loc-Gal80 + p426-Empty | Fig5A |
| och1Δ | p425-Loc-Gal80 + p426-Gal4AD-Cat | Fig5A |
| och1Δ | p425-Loc-Gal80 + p426-Id2-Cat | Fig5A, 6 |
| och1Δ | p425-Loc-Gal11 + p426-Empty | Fig5B |
| och1Δ | p425-Loc-Gal11 + p426-Gal4AD-Cat | Fig5B |
| och1Δ | p425-Loc-Gal11 + p426-Id2-Cat | Fig5B, 6 |
| och1Δ | p425-Loc-Rpt4 + p426-Empty | Fig5C |
| och1Δ | p425-Loc-Rpt4 + p426-Gal4AD-Cat | Fig5C |
| och1Δ | p425-Loc-Rpt4 + p426-Id2-Cat | Fig5C, 6 |
| och1Δ | p425-Loc-Hap5 + p426-Empty | Fig5D |
| och1Δ | p425-Loc-Hap5 + p426-Gal4AD-Cat | Fig5D |
| och1Δ | p425-Loc-Hap5 + p426-Id2-Cat | Fig5D, 6 |
| och1Δ | p425-Loc-Rpt6 + p426-Empty | Fig5E |
| och1Δ | p425-Loc-Rpt6 + p426-Gal4AD-Cat | Fig5E |
| och1Δ | p425-Loc-Rpt6 + p426-Id2-Cat | Fig5E, 6 |
| och1Δ | P425-Loc(stop codon)+p426-Id2-Cat | Fig6 |
| och1Δ | P425-Loc-SV40TAg+p426-Empty | FigS2 |
| och1Δ | P425-Empty+p426-p53-Cat | FigS2 |
| Wild type | P425-Empty + p426-Empty | FigS3 |
| Wild type | P425-Empty + p426-full length *OCH1* | FigS3 |
| Wild type | P425-Empty + p426-Id2-Cat | FigS3 |
| Wild type | P425-Loc-MyoD + p426-Empty | FigS3 |
| Wild type | P425-Loc-MyoD+ p426-Id2-Cat | FigS3 |
| Wild type | P425-Empty+p426-p53-Cat | FigS3 |
| Wild type | P425-Loc-SV40TAg+p426-p53-Cat | FigS3 |
| Wild type | P425-Loc-SV40TAg+p426-Empty | FigS3 |
