# Supplementary material for: A Two-Hybrid Assay to Study Protein Interactions within the Secretory Pathway
Source: PLoS One. 2010 Dec 28;5(12):e15648. doi: 10.1371/journal.pone.0015648 (PMC3011011; doi:10.1371/journal.pone.0015648)
Supplement: Methods S1 — Plasmid construction. Provides detailed description of the methods used for plasmid construction. (DOC) [file pone.0015648.s010.doc]

Supporting Information Methods

**Plasmid construction.** Restriction enzymes, DNA ligase, and calf-intestine phosphatase (CIP) were obtained from New England Biolabs. Cloned Pfu polymerase was obtained from Stratagene. *Escherichia coli* strain DH5α (McLab, South San Francisco, CA) or Top10 (Invitrogen) were used for subcloning. *E. coli* were cultured at 37 °C in LB medium containing 50 μg/mL ampicillin. Plasmids p425-TEF and p426-TEF [1] were obtained from the ATCC (Manassas, VA).

*Full-length OCH1.* We used polymerase chain reaction (PCR) to amplify a 1443-bp fragment encoding *S. cerevisiae* *OCH1* (amino acids 1-481, RefSeq accession number NP_011477.1) and to introduce an SpeI site at the 5’ end of the coding strand and an XhoI site at the 3’ end of the coding strand. This insert was ligated between SpeI and XhoI sites of the plasmid p426-TEF to produce p426-OCH1.

*Localization domain plasmids.*  We used PCR to amplify a 240-bp fragment encoding the localization domain of *S. cerevisiae* *OCH1* (amino acids 1-80, RefSeq accession number NP_011477.1) and used the primers LOCF and LOCR to introduce a SpeI site at the 5’ end of the coding strand and a BamHI site at the 3’ end of the coding strand. This insert was ligated between SpeI and BamHI sites of the plasmid p425-TEF to produce p425-LOC. We then used PCR to amplify a 957-bp fragment encoding murine MyoD (amino acids 1-318, RefSeq accession number NW_001030849) and to introduce restriction sites at the 5’ and 3’ end of the coding strand. We used a primer MyoDF that contained BamHI, AvrII and NdeI sites at the 5’ end of the coding strand and a primer MyoDR that contained XhoI, NheI and SphI at the 3’ end of the coding strand to allow flexibility for future cloning of test proteins into this plasmid. This insert was ligated between BamHI and XhoI sites of p425-LOC to produce p425-LOC-MyoD. A similar strategy was used to create p425-LOC-SV40TAg. For this plasmid, an 1863-bp fragment encoding simian virus 40 large T-antigen (amino acids 88-708, RefSeq accession number NP_043127) was PCR amplified with primers SV40F and SV40R and ligated beween BamHI and XhoI sites of p425-LOC to produce p425-LOC-SV40TAg. For the Gal4p AD project, we used a similar strategy to construct the p425-LOC plasmids with Gal80 (amino acids 1-435, RefSeq accession number NP_013661), Gal11 (amino acids 1-351, RefSeq accession number NP_014591.1), Hap5 (amino acids 1-243, RefSeq accession number NP_015003.1), Rpt6 (amino acids 1-406, RefSeq accession number NP_011467.1), and Rpt4 (amino acids 1-438, RefSeq accession number NP_014902.1). Each gene was cloned into p425-LOC between BamHI and XhoI. For p425-LOC (stop codon) without a fusion protein, we added a stop codon TGA at the end of the reverse primer used to amplify the LOC domain. Primers used are listed in the Supporting Table 1.

*Catalytic domain plasmids.* Since the catalytic domain chimeras contain no localization cues, they require an N-terminal signal peptide to ensure their entry into the secretory pathway. For this purpose we used a prepro sequence that has been shown to direct the high-level, cell surface expression of human proteins in yeast (personal communication, Jennifer Cochran, Stanford University). We used PCR to amplify a 126-bp fragment encoding the prepro sequence (atgaaggttttgattgtcttgttcgctatcttcgctgctttgcccttggctctagc-tcaaccggttatttctactaccgtcggttccgctgcagaaggctctttggacaagagagaagctcggccg; template DNA was a gift from J. Cochran, Stanford University) and to introduce a SpeI site at the 5’ end of the coding strand and a BamHI site at the 3’ end of the coding strand. This insert was ligated between SpeI and BamHI sites of the plasmid p426-TEF to produce p426-prepro. We then used PCR to amplify a 1212-bp fragment encoding the catalytic domain of *S. cerevisiae* *OCH1* (amino acids 78-481, RefSeq accession number NP_011477.1) and to introduce HindIII and XhoI sites at the 5’ and 3’ end of the coding strand, respectively. This insert was ligated between HindIII and XhoI sites of the plasmid p426-prepro to produce p426-CAT. We used PCR to amplify a 291-bp fragment encoding amino acids 38-137 of murine Id2 (RefSeq accession number NM_010496.3) and to introduce restriction sites at the 5’ and 3’ ends of the coding strand. We used a truncated form of Id2 lacking the short N-terminal region that had been shown to interact with Id2’s HLH domain [2]. We used a primer Id2F that contained BamHI and AvrII sites at the 5’ end of the coding strand and a primer Id2R that contained HindIII, NheI and SphI at the 3’ end of the coding strand to allow flexibility for future cloning of test proteins into this plasmid. This insert was ligated between BamHI and HindIII sites of p426-CAT to produce p426-Id2-CAT. A similar strategy was used to create p426-p53-CAT. For this plasmid, a 795-bp fragment encoding murine p53 (amino acids 125-390, RefSeq accession number NM_011640.3) was PCR-amplified with the primers p53F and p53R and ligated between BamHI and HindIII sites of p426-CAT to produce p426-p53-CAT. We used a similar strategy to construct the p426-Gal4AD-CAT (amino acids 841-874, RefSeq accession number NP_015076). The primers Gal4ADF and Gal4ADR were used to PCR-amplify the Gal4AD and the Gal4AD was ligated between BamHI and HindIII into p426-CAT. Primers used to prepare catalytic domain constructs are listed in Supporting Table 2.

*Construction of plasmids encoding MyoD and Id2 point mutants and deletions.* The p425-LOC-MyoD and p426-prepro-Id2-CAT vectors were used as templates for PCR-based mutagenesis using the QuikChange site-directed mutagenesis protocol from Stratagene. PfuTurbo® DNA polymerase (Stratagene) and a standard PCR amplification program were employed. The PCR product was treated with Dpn I endonuclease (New England Biolabs) that specifically digests the methylated DNA template, and selects for the mutation-containing synthesized DNA. We used the same strategy to make deletion mutations using overlap primers. The synthetic oligonucleotides employed are shown in Supporting Table 3.

**Supporting Information References**

1. Mumberg D, Muller R, Funk M (1995) Yeast vectors for the controlled expression of heterologous proteins in different genetic backgrounds. Gene 156: 119-122.

2. Colombo N, Cabrele C (2006) Synthesis and conformational analysis of Id2 protein fragments: Impact of chain length and point mutations on the structural HLH motif. J Peptide Sci 12: 550-558.
